# A case of secondary lymphangiectasia due to ancylostoma intestinal infection

**DOI:** 10.1055/a-2724-6544

**Published:** 2025-11-10

**Authors:** Agostino Cosenza, Luca Elli, Anna Maraschini, Elia Fracas, Lucia Scaramella

**Affiliations:** 1 9304 Department of Pathophysiology and Organ Transplantation, University of Milan, Milan, Italy; 2 9339 Department of Pathophysiology and Organ Transplantation, Fondazione IRCCS Ca’ Granda Ospedale Maggiore Policlinico, University of Milan, Milan, Italy; 3 9339 Laboratory of Microbiology and Virology, Fondazione IRCCS Ca’ Granda Ospedale Maggiore Policlinico, Milan, Italy; 4 9339 Fondazione IRCCS Ca’ Granda Ospedale Maggiore Policlinico, Milan, Italy

A 33-year-old patient presented to an outpatient clinic with a diagnosis of intestinal lymphangiectasia following the onset of dependent edema, hypoalbuminemia, and altered liver transaminases in January/2024. Further evaluation revealed hypogammaglobulinemia, IgA/IgG/IgM deficiency, negative anti-TTG antibodies, normal fecal calprotectin/elastase, and persistent hypoalbuminemia.

The patient was on biweekly human albumin infusions, medium-chain triglyceride (MCT) oil, and vitamin D supplementation.

To further investigate the suspected gastrointestinal pathology, the patient underwent the following diagnostic procedures:

**Esophagogastroduodenoscopy (EGD):** Duodenal bulb/D2 with epithelial disruption. Histology showed normotrophic villi with lymphoplasmacytic/eosinophilic infiltration. *Helicobacter pylori* and Tropheryma whipplei were negative, and intraepithelial T-lymphocyte count (CD3+) was not increased.

**Video capsule endoscopy (VCE):** Diffuse lymphangiectasia in the duodenum/proximal jejunum, pseudopolypoid appearance in some frames, and mild distal villous edema.

**Fig. 1:**
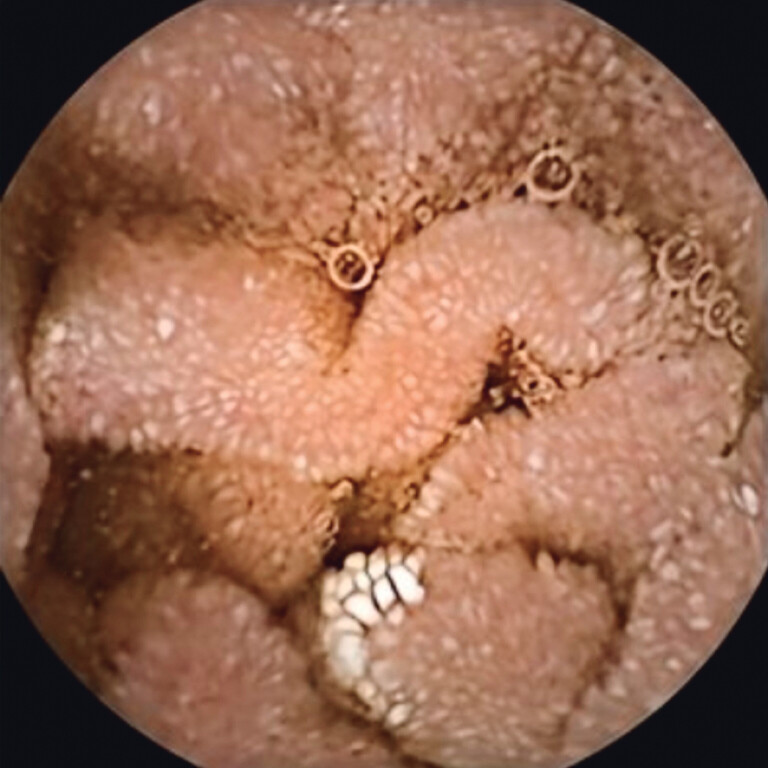
Diffuse edema of the villi with lymphangiectasia in the duodenum.

**Fig. 2:**
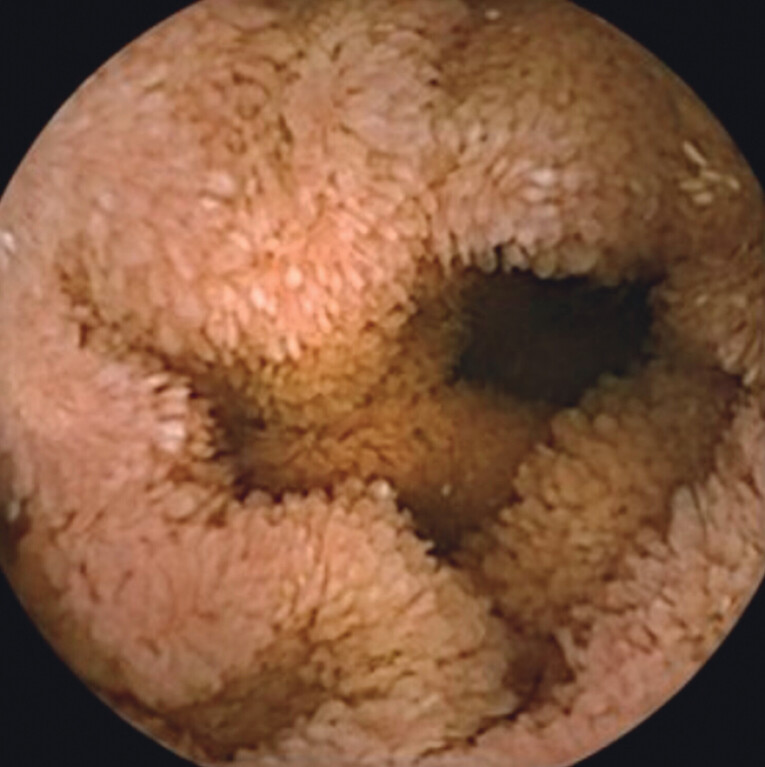
Diffuse edema of the villi in the duodenum.

Due to the persistence of lymphangiectasia with minimal improvement following albumin and MCT oil supplementation, further evaluation of the small intestine was pursued:

**Capsule endoscopy (PillCam SB3, Given Medtronic):** demonstrated mild lymphangiectasia in the proximal and mid-small intestine with mild villous edema ( Fig. 1 and Fig. 2 ).

**Anterograde double-balloon enteroscopy (DBE) (Fujifilm EN-580T):** Diffusely edematous mucosa with shortened, whitish villi, consistent with lymphangiectasia in the explored segments. Presence of two pseudopolyps in the proximal jejunum, which were biopsied, and a vermiform parasite, which was removed ( Video 1 ).

The video shows edematous mucosa with areas of lymphangiectasia in the proximal jejunum, where a motile vermiform parasite and two pseudopolyps are seen; the parasite is retrieved with biopsy forceps.Video 1

**Fig. 3:**
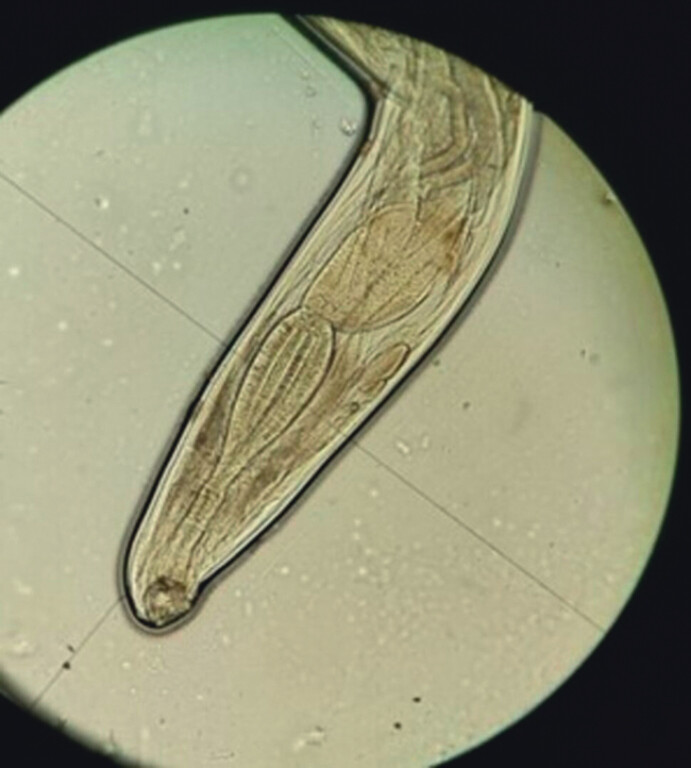
Direct visualization of a male ancylostoma.

**Fig. 4:**
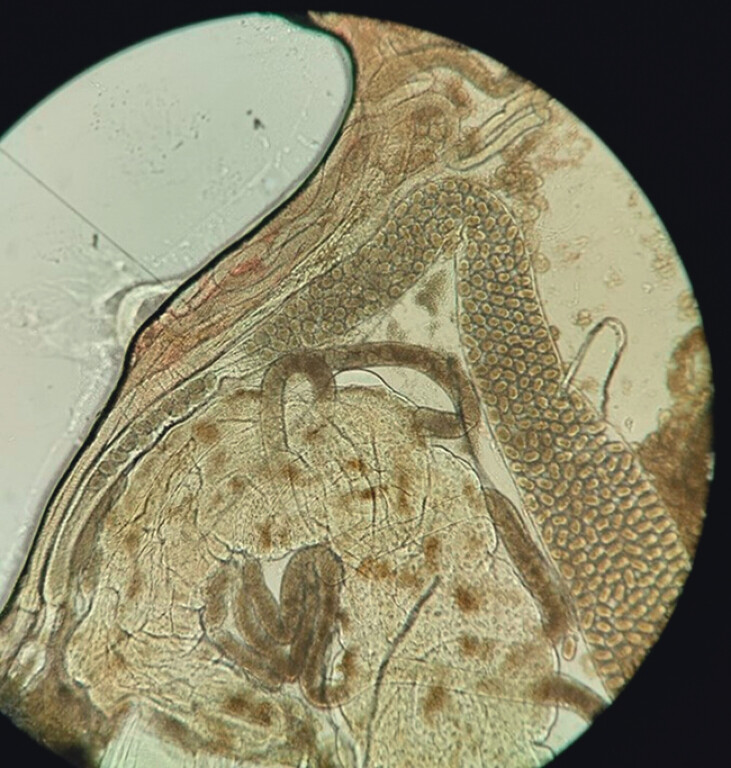
Direct visualization of a female ancylosotma with fertilized eggs.

Histopathological analyses showed no dysplasia in the two pseudopolyps, which exhibited only mild inflammation of the lamina propria. Parasitological analysis identified the specimen as two hookworms of *Ancylostoma spp.* (male and female in reproductive phase) ( Fig. 3 and Fig. 4 ).

Despite negative stool cultures for hookworm (repeated twice), only *Blastocystis hominis* was detected. Infectious disease specialists advised treating *Ancylostoma* with Albendazole (400 mg TID) with a rapid improvement of Immunoglobulin and albumin blood levels.

Endoscopy_UCTN_Code_CCL_1AC_2AG

